# Supplementary material for: COVID-19 health information sources and their associations with preventive behaviors: A typological study with older residents in Seoul, South Korea
Source: PLoS One. 2023 Aug 31;18(8):e0290824. doi: 10.1371/journal.pone.0290824 (PMC10471008; doi:10.1371/journal.pone.0290824)
Supplement: Attachment — Submitted filename: Reviewers report_PONE-D-23-04413.docx [file pone.0290824.s001.docx]

Thank you for the opportunity to review the manuscript titled, “COVID-19 Health Information Sources and Their Associations with Preventive Behaviors in South Korean Older Adults: A Typological Study.” The detailed comments are as follow:

1. p.3: The reason why the authors select Korean older adults as respondents needs to be explained. For example, in terms of the importance of COVID-19 policies or rapid aging etc.
2. P. 4: The authors need to provide more detailed explanation regarding “The advantages of these interpersonal sources include accessibility, trustworthiness, and similarities in individuals’ understandings and beliefs related to health and healthcare [14].” Readers who are not specialized in communication studies may not exactly understand the meaning.
3. P.5: The meaning of “(e.g., restricted sources and usage of health information)” is not very clear.
4. P.5: If the respondents were recruited only in Seoul, it should be acknowledged throughout the manuscript. The expressions such as ‘Korean older adults’ are misleading because Seoul and Korea are not the same. How about changing the title of the article, and revising expressions so that readers clearly know that the study concerns senior citizens in Seoul (not overall older adults in Korea).
5. P.6: Why *female, married, high school education or more* were coded as 2? If these are dummy variables, they should be coded as 0.
6. P.12: The limitation of quota sampling should be mentioned. Quota sampling is not a probability sampling, meaning, the results are not generalizable in principle.
