# Supplementary material for: COVID-19 health information sources and their associations with preventive behaviors: A typological study with older residents in Seoul, South Korea
Source: PLoS One. 2023 Aug 31;18(8):e0290824. doi: 10.1371/journal.pone.0290824 (PMC10471008; doi:10.1371/journal.pone.0290824)
Supplement: Attachment — Submitted filename: response letter.doc [file pone.0290824.s002.doc]

June 28, 2023

Claire Seungeun Lee, Ph.D.
Academic Editor, *PLOS One*

Dear Dr. Lee,

Enclosed is our revision of the manuscript entitled “COVID-19 Health Information Sources and Their Associations with Preventive Behaviors in South Korean Older Adults: A Typological Study” (PONE-D-23-04413). We sincerely appreciate you and the reviewers for the thoughtful comments on our work. This letter addresses how we responded to each of the concerns raised. The revised parts in the manuscript are highlighted.

**Comment from the Editor**

1. Please ensure that your manuscript meets PLOS ONE's style requirements, including those for file naming. The PLOS ONE style templates can be found at

[https://journals.plos.org/plosone/s/file?id=wjVg/PLOSOne_formatting_sample_main_body.pdf](https://urldefense.com/v3/__https:/journals.plos.org/plosone/s/file?id=wjVg*PLOSOne_formatting_sample_main_body.pdf__;Lw!!LIr3w8kk_Xxm!u9Api-EttZJWn5r4Q-V4yiRtUx-enwODOryn_FRvXQm1Egqig6Qk_bZk-gvqJXv-uO4xkHO_Rtm2hGPa$) and

[https://journals.plos.org/plosone/s/file?id=ba62/PLOSOne_formatting_sample_title_authors_affiliations.pdf](https://urldefense.com/v3/__https:/journals.plos.org/plosone/s/file?id=ba62*PLOSOne_formatting_sample_title_authors_affiliations.pdf__;Lw!!LIr3w8kk_Xxm!u9Api-EttZJWn5r4Q-V4yiRtUx-enwODOryn_FRvXQm1Egqig6Qk_bZk-gvqJXv-uO4xkHO_RsmizQBs$)

Response: The manuscript has been re-formatted following the journal requirements.

2. Please provide additional details regarding participant consent. In the ethics statement in the Methods and online submission information, please ensure that you have specified (1) whether consent was informed and (2) what type you obtained (for instance, written or verbal, and if verbal, how it was documented and witnessed). If your study included minors, state whether you obtained consent from parents or guardians. If the need for consent was waived by the ethics committee, please include this information.

If you are reporting a retrospective study of medical records or archived samples, please ensure that you have discussed whether all data were fully anonymized before you accessed them and/or whether the IRB or ethics committee waived the requirement for informed consent. If patients provided informed written consent to have data from their medical records used in research, please include this information

Once you have amended this/these statement(s) in the Methods section of the manuscript, please add the same text to the “Ethics Statement” field of the submission form (via “Edit Submission”).

For additional information about PLOS ONE ethical requirements for human subjects research, please refer to [http://journals.plos.org/plosone/s/submission-guidelines#loc-human-subjects-research](https://urldefense.com/v3/__http:/journals.plos.org/plosone/s/submission-guidelines*loc-human-subjects-research__;Iw!!LIr3w8kk_Xxm!u9Api-EttZJWn5r4Q-V4yiRtUx-enwODOryn_FRvXQm1Egqig6Qk_bZk-gvqJXv-uO4xkHO_RuAyRebl$).

Response: We have included detailed information on the consenting process both in the text and online submission form.

The project was approved by the Institutional Review Board of Ewha Womans University (ewha-202006-0015-01). Prior to the survey, all participants signed on a written consent form.

3. Thank you for stating the following financial disclosure:

“Data collection was supported by the National Research Foundation of Korea (NRF-2020S1A5C2A03092919, PI: Soondool Chung, PhD).”

Please state what role the funders took in the study.  If the funders had no role, please state: "The funders had no role in study design, data collection and analysis, decision to publish, or preparation of the manuscript."

If this statement is not correct you must amend it as needed.

Please include this amended Role of Funder statement in your cover letter; we will change the online submission form on your behalf.

Response: We have included the statement on the funder’s role as below.

Data collection was supported by the National Research Foundation of Korea (NRF-2020S1A5C2A03092919, PI: Soondool Chung, PhD). The funder had no role in study design, data collection and analysis, decision to publish, or preparation of the manuscript.

We have included the statement in the cover letter as well.

4. Thank you for stating the following in your Competing Interests section:

“There are no potential conflicts of interest, and no financial disclosures were reported by the authors of this paper.”

Please complete your Competing Interests on the online submission form to state any Competing Interests. If you have no competing interests, please state "The authors have declared that no competing interests exist.", as detailed online in our guide for authors at [http://journals.plos.org/plosone/s/submit-now](https://urldefense.com/v3/__http:/journals.plos.org/plosone/s/submit-now__;!!LIr3w8kk_Xxm!u9Api-EttZJWn5r4Q-V4yiRtUx-enwODOryn_FRvXQm1Egqig6Qk_bZk-gvqJXv-uO4xkHO_Ri0jCQiH$)

 This information should be included in your cover letter; we will change the online submission form on your behalf.

Response: We have completed the requirement on competing interests on the online submission form.

5. Thank you for stating the following in the Acknowledgments Section of your manuscript:

“Data collection was supported by the National Research Foundation of Korea (NRF-2020S1A5C2A03092919, PI: Soondool Chung, PhD). There are no potential conflicts of interest, and no financial disclosures were reported by the authors of this paper.”

We note that you have provided funding information that is not currently declared in your Funding Statement. However, funding information should not appear in the Acknowledgments section or other areas of your manuscript. We will only publish funding information present in the Funding Statement section of the online submission form.

Please remove any funding-related text from the manuscript and let us know how you would like to update your Funding Statement. Currently, your Funding Statement reads as follows:

“There are no potential conflicts of interest, and no financial disclosures were reported by the authors of this paper.”

Please include your amended statements within your cover letter; we will change the online submission form on your behalf.

Response: We have relocated the information as requested.

6. In your Data Availability statement, you have not specified where the minimal data set underlying the results described in your manuscript can be found. PLOS defines a study's minimal data set as the underlying data used to reach the conclusions drawn in the manuscript and any additional data required to replicate the reported study findings in their entirety. All PLOS journals require that the minimal data set be made fully available. For more information about our data policy, please see [http://journals.plos.org/plosone/s/data-availability](https://urldefense.com/v3/__http:/journals.plos.org/plosone/s/data-availability__;!!LIr3w8kk_Xxm!u9Api-EttZJWn5r4Q-V4yiRtUx-enwODOryn_FRvXQm1Egqig6Qk_bZk-gvqJXv-uO4xkHO_Rooasyru$).

Upon re-submitting your revised manuscript, please upload your study’s minimal underlying data set as either Supporting Information files or to a stable, public repository and include the relevant URLs, DOIs, or accession numbers within your revised cover letter. For a list of acceptable repositories, please see [http://journals.plos.org/plosone/s/data-availability#loc-recommended-repositories](https://urldefense.com/v3/__http:/journals.plos.org/plosone/s/data-availability*loc-recommended-repositories__;Iw!!LIr3w8kk_Xxm!u9Api-EttZJWn5r4Q-V4yiRtUx-enwODOryn_FRvXQm1Egqig6Qk_bZk-gvqJXv-uO4xkHO_Rj9NK9R5$). Any potentially identifying patient information must be fully anonymized.

Important: If there are ethical or legal restrictions to sharing your data publicly, please explain these restrictions in detail. Please see our guidelines for more information on what we consider unacceptable restrictions to publicly sharing data: [http://journals.plos.org/plosone/s/data-availability#loc-unacceptable-data-access-restrictions](https://urldefense.com/v3/__http:/journals.plos.org/plosone/s/data-availability*loc-unacceptable-data-access-restrictions__;Iw!!LIr3w8kk_Xxm!u9Api-EttZJWn5r4Q-V4yiRtUx-enwODOryn_FRvXQm1Egqig6Qk_bZk-gvqJXv-uO4xkHO_RjPlRnNq$). Note that it is not acceptable for the authors to be the sole named individuals responsible for ensuring data access.

We will update your Data Availability statement to reflect the information you provide in your cover letter.

Response: We have provided new data availability statements as below.

Due to ethical concerns, supporting data cannot be openly available. Request for data access should be made through the Ewha Institute for Age Integration Research (<https://cms.ewha.ac.kr/user/indexMain.action?siteId=sskeiairen>).

7. Please review your reference list to ensure that it is complete and correct. If you have cited papers that have been retracted, please include the rationale for doing so in the manuscript text, or remove these references and replace them with relevant current references. Any changes to the reference list should be mentioned in the rebuttal letter that accompanies your revised manuscript. If you need to cite a retracted article, indicate the article’s retracted status in the References list and also include a citation and full reference for the retraction notice.

Response: References have been confirmed, and newly added ones are highlighted.

**Comment from the Reviewer 1**

I believe that this manuscript examines an important issue for older adults in Korea by examining their sources of COVID-19 health information and preventive behaviors during the COVID-19 pandemic. The authors have employed appropriate methods to address their research questions. However, it is necessary for the authors to provide Korean context and background information regarding older adults and the COVID-19 pandemic. Additionally, a more comprehensive literature review of prior studies on information sources and preventive behaviors among Koreans is required. Including articles such as Jang, S.H. (2022) "Social-ecological factors related to preventive behaviors during the COVID-19 pandemic in South Korea" and Jang, S.H. (2022) "Disparities in COVID-19 Information Sources and Knowledge in South Korea" would be beneficial. Moreover, the discussion section should incorporate policy implications considering the specific South Korean context.

Response: We have included contextual information about the target population and the COVID-19 pandemic and added relevant references on previous studies on the topic. We sincerely appreciate the reviewer for recommending relevant studies.

South Korea has been recognized for its rapid and effective response to the outbreak of COVID-19, and the Korean government has proactively implemented policies and services to control the spread of virus and disseminate COVID-19 information [6–8]. Previous studies with Koreans have addressed various impacts posed by the COVID-19 pandemic; however, attention has rarely been paid to the sources of health information particularly among older individuals [8, 9].

The authors should review the inconsistency in the definition of older adults used throughout the manuscript (65+ vs. 60 years old; p.5) and ensure that the inclusion and exclusion criteria are clearly stated and adhered to consistently. Furthermore, it would be advantageous for the authors to provide a rationale for focusing on older adults and to compare information sources and preventive behaviors between older adults and their younger counterparts based on earlier studies.

Response: The inconsistent report of the lower end of age range has been corrected, and we have added statements that rationalize our focus on older adults as below.

Given the age-associated vulnerability to health and social risks (e.g., physical health challenges, mental health problems, social isolation) and knowledge gap (e.g., misinformation, barriers to health information acquisition) during the pandemic [3, 4], the present study focuses on older individuals.

The focus on older adults was prompted by their disadvantages in accessing health information particularly through digital sources [3, 15–18].

Regarding the selection of 12 information sources, it would be helpful if the authors provided references or a rationale for their choices.

Response: Although there was no original source of the measure, we have reviewed different types of information sources in the introduction section and cited previous studies using a similar set of information sources.

As primary means of mass communication, newspapers, magazines, television, and radio are conventional sources of health information for many older adults [9], including older Koreans [10]. With advances in communication and information technologies, the Internet and social networking sites have become a popular source of health information as well, with increasing use in older populations [11, 12]. Network members (e.g., family, friends, co-workers) are also traditional sources of health information for older adults [13]. These interpersonal sources are not only easy to access but also trustworthy because they represent social conveys with familiarity [13,14]. The credibility of sources is also an important factor, because people often turn to health professionals or authorities for critical health needs [15, 16].

Similar to recent studies on health information sources [9, 13, 17, 18], the list included newspapers, magazines, television, radio, Internet, social networking sites, family members, friends, former/current coworkers, healthcare professionals, government/disease control centers, and the World Health Organization.

Given the variation in neighborhood socioeconomic status (SES) and COVID-19 confirmed rates within Seoul, it is suggested that the authors include the district of Seoul (e.g., Gangnam-gu, Dobong-gu) as a control variable.

Overall, addressing these points will enhance the manuscript by providing a clearer contextual understanding, a more thorough literature review, and additional considerations for policy implications and control variables.

Response: Following the reviewer’s suggestion, we have added regions as a covariate. The districts were categorized into 4 areas. Although region was not a significant predictor in the multivariate model, there was a regional difference in the typology. The finding is now discussed along with its policy implications.

Based on the district of residence, region in Seoul was classified into four geographic areas (0 = northeast, 1 = northwest, 3 = southeast, 4 = southwest).

Participants’ area of residence was widely spread across the city of Seoul (33% in northeast areas, 18.5% in northwest areas, 19% in southeast areas, and 29.5% in southwest areas).

Given that a notably high proportion of the *limited* group members were residents in the northeast part of Seoul (e.g., Kangbuk gu, Nowon gu, Dobong gu) where area socioeconomic disparities are known [26], more resources and services should be allocated for disseminating COVID-19 health information in these areas.

**Comment from the Reviewer 2**

1. p.3: The reason why the authors select Korean older adults as respondents needs to be explained. For example, in terms of the importance of COVID-19 policies or rapid aging etc.

Response: We have justified our focus on the target population and provided contextual information as indicated in our earlier response.

2. p.4: The authors need to provide more detailed explanation regarding “The advantages of these interpersonal sources include accessibility, trustworthiness, and similarities in individuals’ understandings and beliefs related to health and healthcare [14].” Readers who are not specialized in communication studies may not exactly understand the meaning.

Response: we have revised the statements as below.

**Network members (e.g., family, friends, co-workers) are also traditional sources of health information for older adults [13]. These interpersonal sources are not only easy to access but also trustworthy because they represent social conveys with familiarity [13,14].**

3. p.5: The meaning of “(e.g., restricted sources and usage of health information)” is not very clear.

Response: To clarified the meaning, the phase has been revised as below.

**restricted sources and limited usage of health information**

4. p.5: If the respondents were recruited only in Seoul, it should be acknowledged throughout the manuscript. The expressions such as ‘Korean older adults’ are misleading because Seoul and Korea are not the same. How about changing the title of the article, and revising expressions so that readers clearly know that the study concerns senior citizens in Seoul (not overall older adults in Korea).

Response: Following the reviewer’s suggestion, our title has been changed to “COVID-19 Health Information Sources and Their Associations with Preventive Behaviors: A Typological Study with Older Residents in Seoul, South Korea.” We sincerely appreciate the reviewer for bringing our attention to the matter.

5. p.6: Why female, married, high school education or more were coded as 2? If these are dummy variables, they should be coded as 0.

Response: Coding information has been revised as below.

Sociodemographic variables included age (in years), gender (0 = *male*, 1 = *female*), marital status (0 = *not married*, 1 = *married*), and education (0 = *less than a high school education*, 1 = *high school education or more*). Participants were also asked to rate their financial status and health status on a 5-point scale from 1 = *very poor* to 5 = *excellent*. Based on the district of residence, region in Seoul was classified into four geographic areas (0 = northeast, 1 = northwest, 3 = southeast, 4 = southwest).

6. p.12: The limitation of quota sampling should be mentioned. Quota sampling is not a probability sampling, meaning, the results are not generalizable in principle.

Response: Limited generalizability associated with quota sampling has been added.

First, its quota sampling methods and cross-sectional design preclude generalization of the finding to a larger population and causal inferences of relationships between a typology of information sources and preventive behaviors.

We believe that our manuscript is substantially improved by these revisions. We hope that you will find it suitable for publication in ***PLOS One.***

Sincerely yours,
